# Mediating effects of DNA methylation in the association between sleep quality and infertility among women of childbearing age

**DOI:** 10.1186/s12889-023-16681-w

**Published:** 2023-09-15

**Authors:** Ying Tang, Hong Gan, Baolin Wang, Xiaorui Wang, Mengdie Li, Qianhui Yang, Menglong Geng, Peng Zhu, Shanshan Shao, Fangbiao Tao

**Affiliations:** 1 Department of Maternal, Child and Adolescent Health, School of Public Health, Anhui Medical University, No. 81 Meishan Road, Hefei, 230032 Anhui China; 2 MOE Key Laboratory of Population Health Across Life Cycle, No 81 Meishan Road, Hefei, 230032 Anhui China; 3 NHC Key Laboratory of Study On Abnormal Gametes and Reproductive Tract, No 81 Meishan Road, Hefei, 230032 Anhui China; 4 Anhui Provincial Key Laboratory of Population Health and Aristogenics, Anhui Medical University, No 81 Meishan Road, Hefei, 230032 Anhui China

**Keywords:** DNA methylation, Epigenetics, Infertility, Ovulation disorders, Sleep quality

## Abstract

**Background:**

This study aims to investigate the association between sleep quality and infertility among women and to explore the mediating effects of DNA methylation in this association.

**Methods:**

This study is a population-based case–control study. The relationship between sleep quality and infertility was investigated in women with anovulatory infertility (*n* = 43) and healthy controls (*n* = 43). Genome-wide DNA methylation was profiled from peripheral blood samples using the Illumina Infinium Human Methylation 850k BeadChip. Differentially methylated CpGs between cases and controls were identified using the *ChAMP* R package. The mediating effect of DNA methylation between sleep quality and infertility among women was investigated using the Bayesian estimation method provided by the R package “mediation”.

**Results:**

The survey included 86 women of reproductive age, with 43 participants each in the case and control groups. The average age of the women was 27.6 ± 2.8 years (case group: 27.8 ± 3.0 years, control group: 27.4 ± 2.7 years). A total of 262 differentially methylated CpGs corresponding to 185 genes were identified. Difficulty falling asleep was a risk factor for infertility in women (*OR* = 3.69, 95%*CI* = 1.14, 11.99), and a causal mediation effect of DNA methylation CpGs was found. The mediating effect coefficient for cg08298632 was 0.10 (95%*CI* = 0.01–0.22), and the proportion of the total effect mediated by this methylation site increased to 64.3%.

**Conclusion:**

These results suggest that DNA methylation CpGs (cg08298632) play a significant role in the relationship between difficulty falling asleep and infertility in females. These findings contribute to our understanding of the underlying mechanisms that connect difficulty falling asleep and infertility in women. Further studies are necessary to fully understand the biological significance and potential therapeutic applications of these findings. The identified DNA methylation sites provide new and valuable insights and potential targets for future studies aiming to prevent and treat female infertility.

**Supplementary Information:**

The online version contains supplementary material available at 10.1186/s12889-023-16681-w.

## Background

Reproductive health plays an important role in human survival, reproduction, and social development. Infertility is one of the most important components of reproductive health. Infertility is diagnosed when a couple is unable to conceive after engaging in unprotected sexual intercourse for one year [1]. In recent years, there has been an increase in the number of individuals seeking infertility treatment [2]. Infertility is a global medical and social concern, with approximately half of the cases attributed to female factors [3]. It not only raises the risk of mental illness, pregnancy-related complications, and chronic diseases but also increases the chances of offspring developing conditions such as mental retardation [4–6]. Moreover, infertility can cause discrimination, strain in marital relationships, domestic violence, and even marital breakdowns [7–9].

Advancements in science and technology have improved the accessibility and effectiveness of infertility treatments. However, these treatments are often expensive, yield unsatisfactory outcomes, and can have side effects such as ovarian hyperstimulation syndrome [10]. Thus, it is necessary to strengthen the surveillance of infertility issues, identify preventable causes of infertility, and tailor effective prevention interventions to reduce the prevalence of this condition.

Infertility is a complex condition influenced by various factors, such as genetics, epigenetics, environmental factors, and lifestyle choices [11–13]. Recently, increased attention has been paid to the adverse effects of sleep behavior and circadian disturbances on fertility. Studies have shown that sleep patterns, such as staying up late and sleeping in on weekends, can lead to social jet lag and sleep disorders, which may adversely affect follicle development and hormone production and increase the risk of pregnancy-related difficulties [14, 15]. Both the US National Health Interview Survey consisting of 9 137 women of childbearing age and a study conducted in China with 2 687 women of childbearing age showed a U-shaped association between sleep duration and the probability of achieving pregnancy [16]. Furthermore, a prospective cohort study found that women with medically diagnosed non-apneic sleep disorders had a 3.7-fold increased risk of infertility compared to those without sleep disorders [17]. The North American pre-pregnancy cohort study also reported reduced fertility among women who experienced nighttime sleep difficulties or insufficient sleep [18].

Although the adverse effects of sleep quality on female reproductive health, such as irregular menstruation, low fertility, infertility, and adverse pregnancy and birth outcomes, have been widely reported, the underlying mechanisms behind these effects have not been fully elucidated. With advances in epigenetics, the association between epigenetic modification and reproductive health has attracted increasing attention [19, 20]. Epigenetic modifications are influenced by a combination of genetic and environmental factors, and DNA methylation is one of the most common and important epigenetic modifications regulating gene expression [21, 22]. Numerous studies have shown that DNA methylation is significantly affected by sleep, and specific genomic regions undergo changes in DNA methylation profiles following sleep deprivation [23–26]. Previous studies have elucidated that aberrant DNA methylation may play an important role in the pathogenesis of conditions such as polycystic ovary syndrome (PCOS) and endometriosis (EMS), and that these defects in DNA methylation can promote the dysregulation of genes involved in inflammation, immunity, and hormone synthesis [27, 28]. Therefore, epigenetic mechanisms, such as DNA methylation, may play an important role in the relationship between sleep quality and infertility in women.

Therefore, this study aimed to clarify the association between sleep quality and infertility among women, and to use causal mediation analysis to further explore the potential mediating effect of DNA methylation on the relationship between sleep quality and infertility. By elucidating the relationship between sleep quality, DNA methylation, and infertility, this study aimed to provide relevant strategies to support the prevention of female infertility.

## Methods

### Study design and population

This study utilized a prospective pre-pregnancy cohort known as the Reproductive Health of Childbearing Couples—Anhui Cohort (RHCC-AC). The RHCC-AC was divided into two subcohorts: newlyweds cohort and infertility-specific cohort. The details of the newlyweds and infertility-specific cohort are described in Table S1. Written informed consent was obtained from each participant prior to enrolment in the study.

**Fig. 1:**
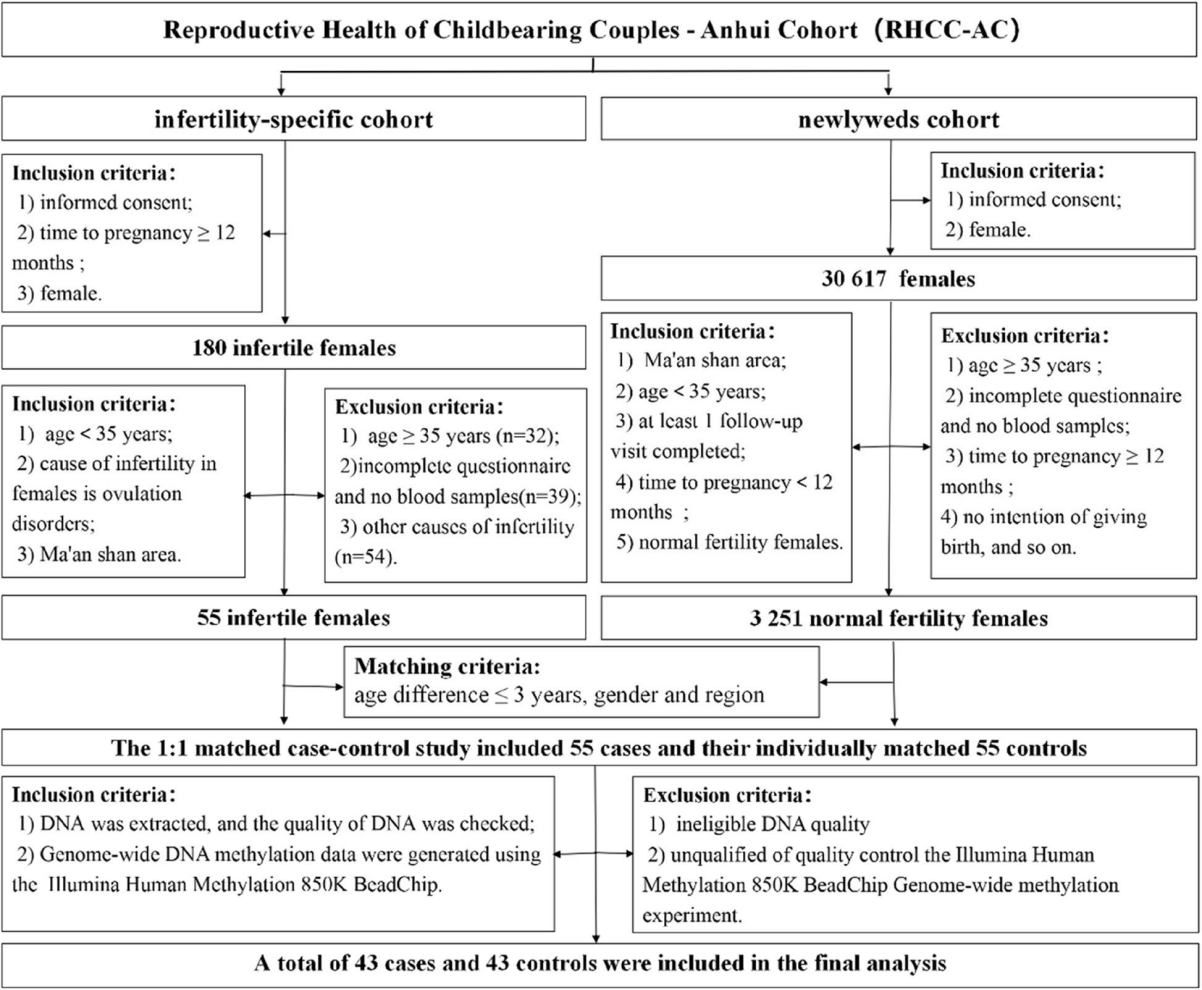
Flowchart depicting the study design

The present study is a population-based, case‒control study involving women with infertility from the infertility-specific cohort as the case group and individually matched women with normal fertility from the newlyweds cohort as the control group. The cases were filtered based on the following exclusion and inclusion criteria. The inclusion criteria were as follows: 1) women aged < 35 years; 2) availability of complete questionnaire data; 3) eligible DNA samples extracted from whole blood samples; 4) failure to achieve a clinical pregnancy after at least 12 months of regular unprotected intercourse; and 5) ovulation disorders identified as the cause of infertility. The exclusion criteria were as follows: 1) other causes of infertility, such as chromosomal anomalies; blocked, damaged, or absent fallopian tubes; EMS, and others and 2) failure to meet the quality control standards for the Illumina Human Methylation 850K BeadChip Genome-wide methylation experiment. Cases and controls were matched in a 1:1 ratio based on age less than or equal to 3 years, sex, and region of residence. A total of 43 cases and 43 controls were included in the final analysis. The flowchart depicting the selection process is shown in Fig. 1.

The Ethics Committee of Anhui Medical University (number: 20189999) approved all study protocols, and informed consent was obtained from all participants before enrolment in the study.

### Information and sample collection

Under the guidance of trained staff, participants completed a self-reported questionnaire that solicited information regarding age (21–25, 26–29, and 30–32 years), education (technical *secondary school or below* or *college degree or above*), income (*less than 60,000 Yuan* or *at least 60,000 Yuan*), employment (*employed* or *unemployed*), menarche age (< *13, 13–15, or* ≥ *15 years*), history of adverse pregnancy (*spontaneous abortion*, *embryo arrest*, *stillbirth, *etc*.*), cigarette smoking (*yes or no*), alcohol consumption (*yes* *or* *no*), consumption of cured/grilled/fried food (≤ *3 times a week or* > *3 times a week*), consumption of sugar-sweetened beverages (SSBs) (≤ *3 times a week or* > *3 times a week*), consumption of takeaway foods (≤ *3 times a week or* > *3 times a week*), average sitting time ([sitting time on weekdays × 5) + (sitting time on rest days × 2])/7, categorized as < *8 h or* ≥ *8 h*], physical activity (based on the International Physical Activity Questionnaire [29], categorized as *moderate or high level of physical activity or low level of physical activity*), duration of electronic device usage before bedtime (< *30 min and* ≥ *30 min*), height and weight, and depressive symptoms.

The study utilized a comprehensive approach to construct two composite variables, namely “socioeconomic status (SES)” and “unhealthy lifestyle.” “SES” was constructed by amalgamating education, income, and employment factors [30], whereas “unhealthy lifestyle” was constructed by amalgamating cigarette smoking; consumption of alcohol, cured/grilled/fried food, SSBs, and takeaway foods; average sitting time; physical activity; and electronic device usage before bedtime [30].

Body mass index (BMI) was calculated using the participants’ weight in kilograms divided by the height in meters squared using the Chinese criteria. The BMI values were then classified into two groups: “underweight and normal weight” (< 23.9 kg/m^2^) and “overweight and obesity” (≥ 24 kg/m^2^). Depressive symptoms were evaluated using the Patient Health Questionnaire 9 (PHQ-9). Further details regarding the evaluation of depressive symptoms are described elsewhere [31].

Clinical information of the study participants, including the type of infertility and the duration for which they were trying to become pregnant, was obtained from their medical records.

### Sleep behaviors

Difficulty falling asleep was assessed using a single question, “How long do you need to fall asleep?” Participants who reported a time of 30 min or longer to fall asleep were classified as experiencing difficulty falling asleep.

Daytime sleepiness among couples of childbearing age was measured using the Epworth Sleepiness Scale (ESS), which was designed at Epworth Hospital in Melbourne, Australia. It serves as a tool to assess the degree of daytime sleepiness easily and accurately among study participants [32]. The ESS consists of eight items, and participants rate individual items on a 4-point scale, with 0 (never doze), 1 (rarely doze), 2 (sometimes doze), and 3 (often doze) points. The scores for the eight items are added up to generate a total score ranging from 0 to 24, with higher scores indicating a more obvious tendency toward daytime sleepiness. According to the scores, participants can be divided into two groups: nondaytime sleepiness (0–10 points) and daytime sleepiness (11–24 points). In this study, the Cronbach’s α coefficient of the ESS was 0.76, indicating good internal consistency.

### Whole blood DNA extraction and BeadChip DNA methylation assay

At the time of participant recruitment, whole blood specimens were collected by the laboratory physician. The samples were stored at room temperature, transferred to the laboratory, and stored in the laboratory at − 80°C until further analysis. Whole blood DNA was extracted and purified from the blood samples of patients and controls using the MagNA Pure nucleic acid purification platform (Roche Diagnostics GmbH, Germany) along with the MagNA Pure 24 nucleic acid purification kit. DNA samples were selected based on an OD260/280 ratio ranging from 1.7 to 2.0 for further experiments. The integrity of all DNA samples was assessed by agarose gel electrophoresis. During electrophoresis, the DNA samples were separated based on size and observed for the presence of a clear main band. The main band was required to be intact and not less than 10 kb in size, and without any obvious degradation.

DNA isolation and analysis of DNA methylation patterns were performed using the Infinium Human Methylation 850 K BeadChips (Illumina Inc., San Diego, CA, USA), which enable the assessment of methylation levels at 853 307 CpG sites across the genome. To prepare the DNA samples for analysis, they were subjected to bisulfite treatment using the EZ DNA Methylation Kit (Zymo Research, Irvine, CA, USA) following the manufacturer’s protocol. The processed DNA samples were then hybridized to the BeadChip (Illumina) according to the Illumina Infinium HD Methylation Protocol. The resulting Illumina intensity data (IDAT) files from the chip were further processed by the R/Bioconductor (version 3.3.3) package ChAMP. Batch effect were adjusted for DNA methylation. To mitigate the potential influence of varying proportions of cell types on DNA methylation patterns [33], methylation data were corrected for cell type heterogeneity between samples [34]. Additionally, we identified differently methylated probes (DMPs) at significance of *BH*-adjusted *p*-value < 0.05 and absolute value of *△β* > 0.05. The DNA methylation level at each CpG site was reported as a* β* value, ranging from 0–1, where 0 represents a nonmethylated probe and 1 represents a fully methylated probe.

### Statistical analysis

All statistical analyses were performed using SPSS 25.0 (SPSS Inc., Chicago, IL, USA), and R 4.1.2 software. A two-sided *P* value < 0.05 was considered statistically significant.

Descriptive statistics, such as mean (standard deviation) or percentage, were used to describe the demographic characteristics of the participants. The chi-square test was used to compare various basic characteristics (e.g., age, SES, lifestyle, depressive symptoms) between the cases and controls. Conditional logistic regression analysis was performed to investigate the effect of sleep characteristics of women on infertility.

The mediator variable, DNA methylation sites, underwent initial screening prior to conducting the causal mediation analysis. The screening process involved three steps. Firstly, the DMP function of the R package ChAMP was utilized to identify methylation sites that exhibited significant differences between the infertility group and the control group. Secondly, the DMP function was employed to identify methylation sites that showed statistical associations with difficulty falling asleep. Thirdly, the candidates identified from the previous two steps, which demonstrated co-associations with both difficulty falling asleep and infertility, were further screened. Finally, the causal mediation effects of the candidate methylation sites were analyzed using the Bayesian estimation method provided by the R package “mediation”. Specifically, the average direct effect, average causal mediation effect, and total effect were estimated.

## Results

### Characteristics of participants

A total of 86 women of reproductive age were included in the survey, with 43 women each in the case and control groups. The average age of the participants was 27.6 ± 2.8 years, and the average ages of the women in the case group and the control group were 27.8 ± 3.0 years and 27.4 ± 2.7 years, respectively. No significant differences were observed in the average age between the two groups (*t* =  − 0.*69, P* > 0.05). The results of the *χ*^2^ goodness-of-fit test indicated that the rates of overweight and obesity in the case group were significantly different from those in the control group (*P* < 0.05; Table 1).

**Table 1:** Basic characteristics of the study subjects

Characteristic	Total population (*n* = 86)	Control group (*n* = 43)	Case group (*n* = 43)	*χ*^*2*^ value	*φ* value
Age (years)				0.93	0.10
21–25	19 (22.1)	10 (52.6)	9 (47.4)		
26–29	43 (50.0)	23 (53.5)	20 (46.5)		
30–32	24 (27.9)	10 (41.7)	14 (58.3)		
BMI (kg/m^2^)				10.81^**^	0.35^**^
< 23.9	60 (69.8)	37 (61.7)	23 (38.3)		
≥ 24	26 (30.2)	6 (23.1)	20 (76.9)		
Socioeconomic status				0.47	0.07
Low	23 (26.7)	11 (47.8)	12 (52.2)		
Medium	45 (52.3)	24 (53.3)	21 (46.7)		
High	18 (20.9)	8 (44.4)	10 (55.6)		
Menarche age (years)				3.15	0.19
< 13	37 (43.0)	16 (43.2)	21 (56.8)		
13–15	36 (41.9)	22 (61.1)	14 (38.9)		
≥ 15	13 (15.1)	5 (38.5)	8 (61.5)		
History of bad pregnancy				0.07	0.03
No	69 (80.2)	35 (50.7)	34 (49.3)		
Yes	17 (19.8)	8 (47.1)	9 (52.9)		
Unhealthy lifestyle scores				0.52	0.08
0	25 (29.1)	11 (44.0)	14 (56.0)		
1	34 (39.5)	18 (52.9)	16 (47.1)		
≥ 2	27 (31.4)	14 (51.9)	13 (48.1)		
Depressive symptoms				0.05	0.03
No	57 (66.3)	29 (50.9)	28 (49.1)		
Yes	29 (33.7)	14 (48.3)	15 (51.7)		

Note: Data presented as n (%)

*Abbereviation: BMI* body mass index

^**^P < 0.01

### Overall changes in blood genomic DNA methylation among women of reproductive age

**Fig. 2:**
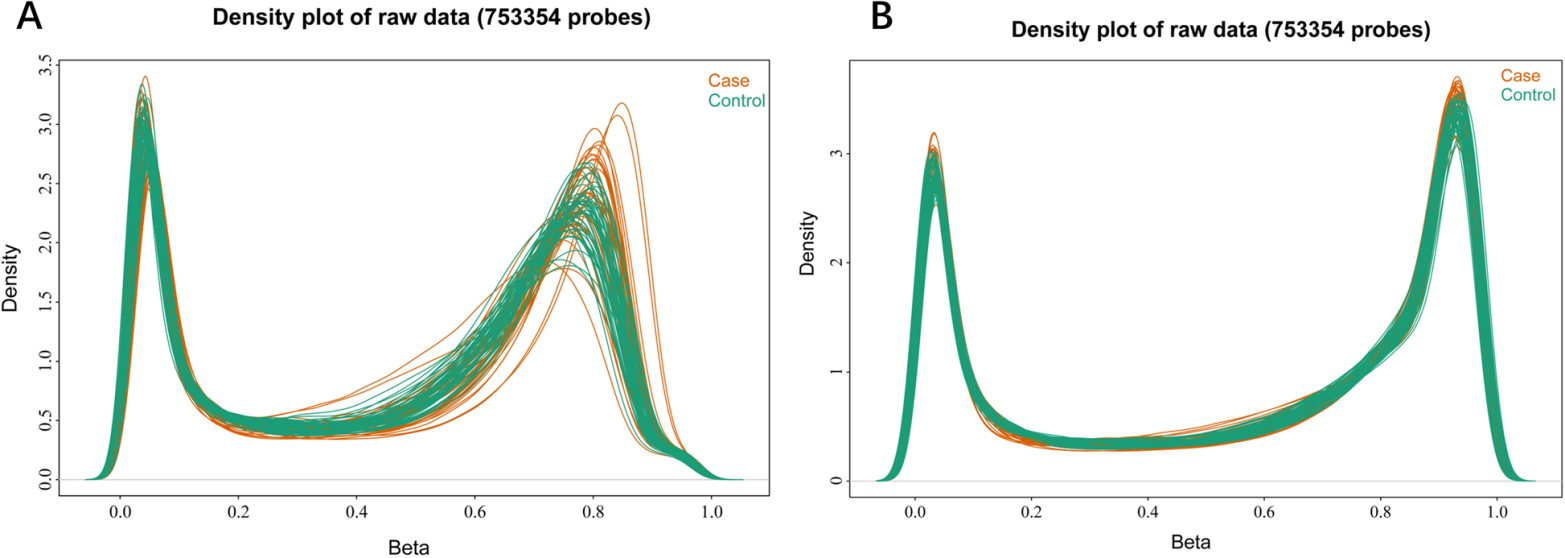
Density curve distribution chart of all samples

**Fig. 3:**
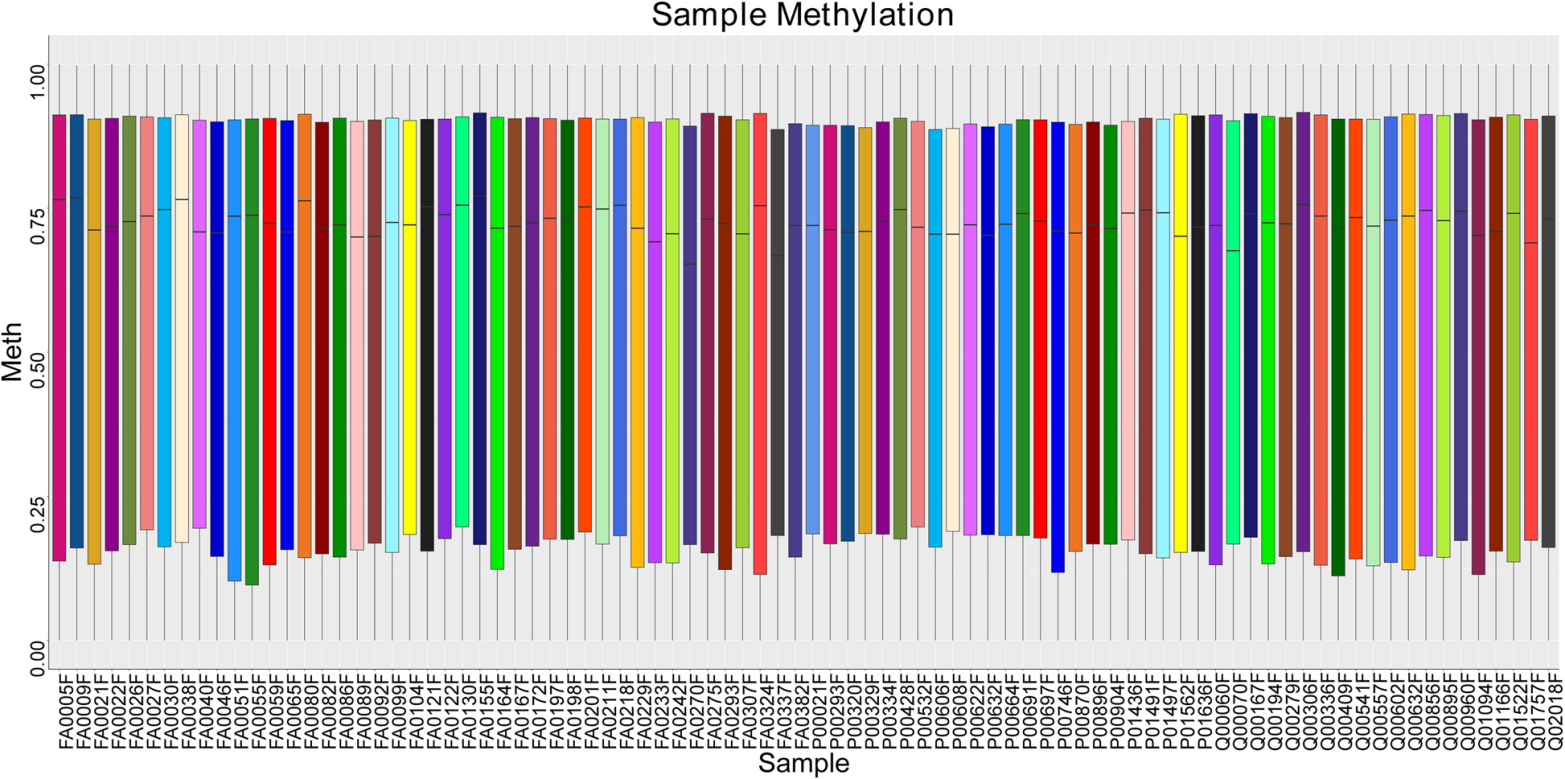
Box plot for case and control samples. The horizontal coordinates in the box-and-line plot represent each sample, the vertical coordinates represent the β-value (a β-value of 0 indicates that no methylation has occurred at the locus, a β-value < 0.2 indicates hypomethylation, a β-value between 0.2 and 0.8 indicates partial methylation, a β-value > 0.8 indicates hypermethylation, and a β-value equal to 1 indicates complete methylation), and the black line in the box represents the median (i.e., the average level of methylation) methylation level. The upper and lower limits of the boxes represent the quartiles of methylation

After data processing and quality control, DNA methylation data generated using the Illumina Methylation EPIC array were available for analysis in 43 cases and 43 controls, and a total of 753 354 eligible CpG sites were selected for further analysis. Figure 2A and B depict the methylation density distribution plots for the original and normalized data, respectively. These plots provide an overview of the distribution of methylation levels across all loci in each sample. The x-axis represents the *β*-value, which ranges from 0 to 1, whereas the y-axis represents the frequency of loci occurrence. Based on the observations presented in Fig. 2, it is evident that the implementation of standardization treatment effectively mitigated the disparities among the samples within each group. Moreover, the overall magnitude of methylation differences between the samples was relatively modest. Furthermore, the density curves exhibited a bimodal distribution, suggesting that the majority of methylated loci were either hypomethylated or hypermethylated. The distribution of average methylation levels for the case and control groups is shown in Fig. 3. The *β*-value, ranging from 0 to 1, represents the methylation level at each CpG site. The evenly distributed boxes in the figure indicate the symmetry of the methylation data in this assay.

**Fig. 4:**
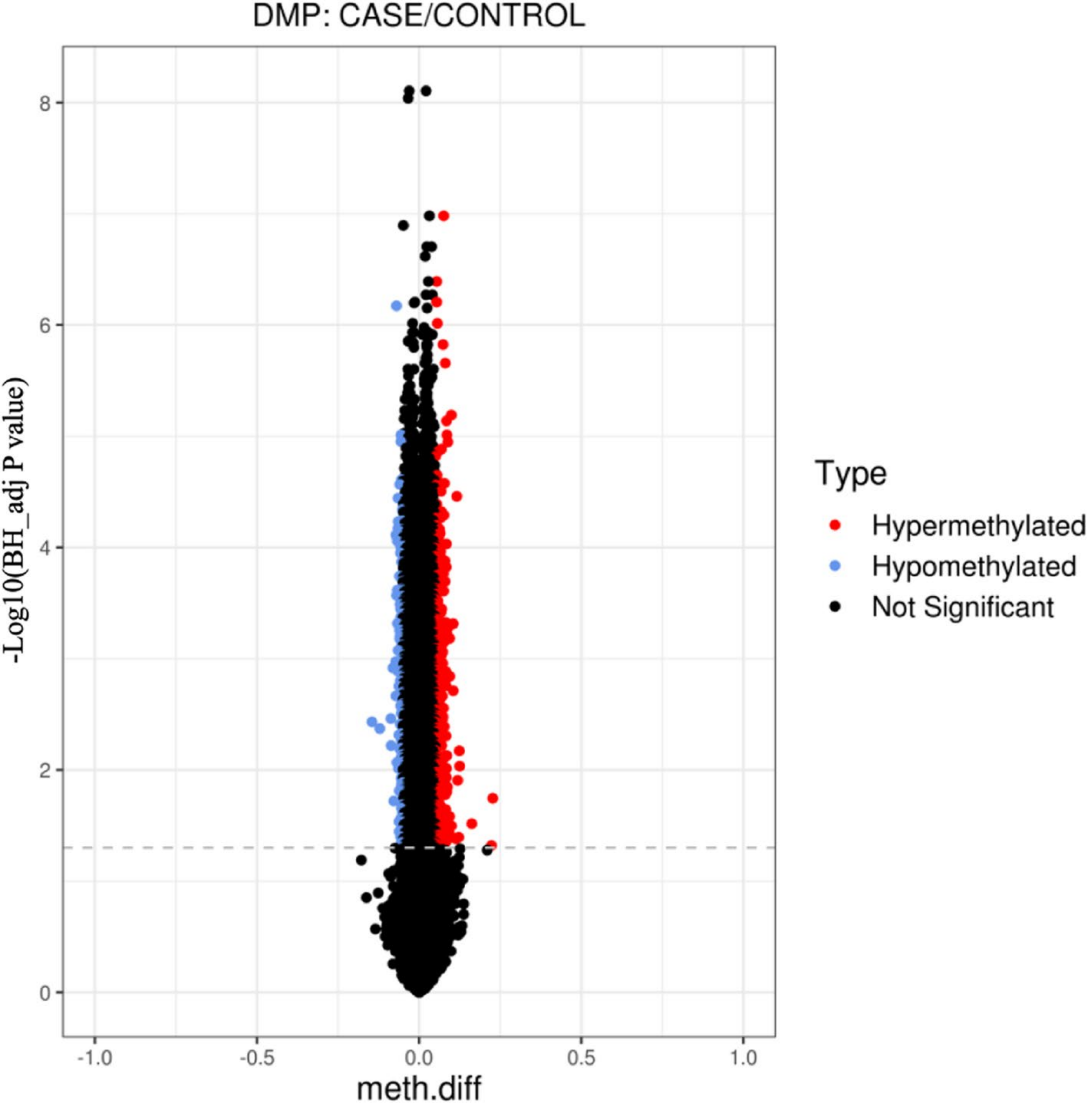
Volcano plot for differential DNA methylation analysis between the case and control groups. The x-axis shows the DNA methylation difference (*△β*), and the y-axis shows the − log^10^ *p*-value of each CpG site. Red color represents hypermethylation sites, blue color represents hypomethylation sites, and black color represents nonsignificant sites

Of the 753 354 probes analyzed on the EPIC BeadChips, a total of 262 differentially methylated CpGs corresponding to 185 differential methylation genes were identified after adjusting for batch effect and cell type effects. Among these, there were 180 hypermethylated CpGs and 82 hypomethylated CpGs (Table S1). Probes that showed a greater than 5% change in *β*-value (*BH*-adjusted *P* < 0.05) between the case and control groups were classified as differentially methylated sites (Fig. 4).

### Effects of sleep quality on female infertility

Among the 86 women of childbearing age included in this study, 26 (30.2%) reported experiencing difficulty falling asleep. The results showed that there were significant differences between the case and control groups in terms of difficulty falling asleep (*χ*^2^ = 5.51, *P* < 0.01). Moreover, after controlling for confounding factors, logistic regression analysis revealed that difficulty falling asleep was a significant risk factor for infertility in women (*OR* = 3.69, 95%*CI* = 1.14, 11.99; Table 2).

**Table 2:** The relationship between sleep quality and infertility of female

Sleep characteristics	Total [n(%)]	Control group [n(%)]	Case group [n(%)]	*χ*^2^-value	*OR* (95%*CI*)^a^
Difficulty falling asleep				5.51^*^	
No	60 (69.8)	35 (58.3)	25 (41.7)		1.00
Yes	26 (30.2)	8 (30.8)	18 (69.2)		3.69 (1.14, 11.99)^*^
Daytime sleepiness				0.82	
No	56 (65.1)	26 (46.4)	30 (53.6)		1.00
Yes	30 (34.9)	17 (56.7)	13 (43.3)		0.45 (0.15, 1.38)

^a^Adjusting age, SES, unhealth lifestyle, menarche age, history of adverse pregnancy, BMI, depressive symptoms

Abbreviations: *OR* Odds ratios, *CI* confidence intervals

^*^P < 0.05

### Causal mediation analysis of difficulty falling asleep, DNA methylation, and infertility among women

**Fig. 5:**
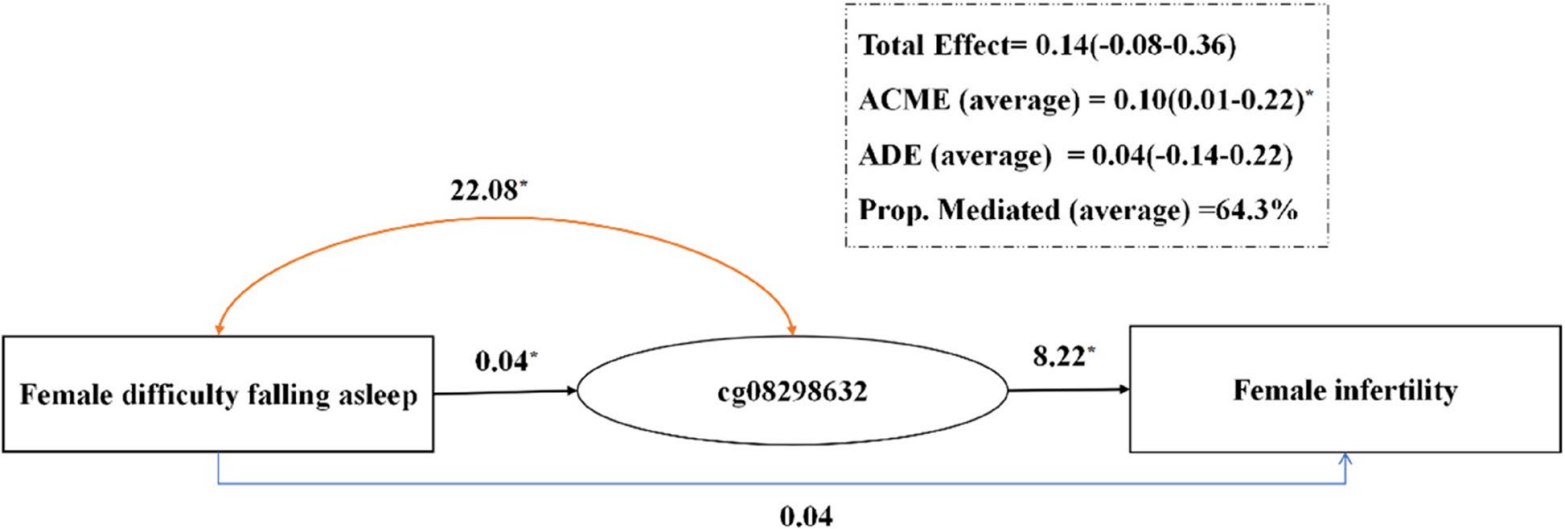
Assessment of the mediating role of DNA methylation in the effect of difficulty falling asleep on infertility among women

The mediator variable, DNA methylation sites, underwent initial screening prior to conducting causal mediation analysis. The DMP function of the R package *ChAMP* was utilized to identify a total of 262 CpG methylation sites that exhibited significant differences between the infertility group and the control group. Eight methylation sites were selected based on their co-association with difficulty falling asleep and infertility. These eight selected CpG sites were included in the causal mediation analysis (Table 3). Model 1, which did not control for covariates, identified only one methylation site, cg08298632, that played a positive mediating role in the relationship between difficulty falling asleep and infertility among women. The mediating effect coefficient for cg08298632 was 0.10 (95%*CI* = 0.01–0.21), and the proportion of the total effect mediated by this methylation site was 37.3% (Table 3). After adjusting for covariates in Model 2, the positive mediating role of cg08298632 in the relationship between difficulty falling asleep and infertility remained. The mediating effect coefficient for cg08298632 was 0.10 (95%*CI* = 0.01–0.22), and the proportion of the total effect mediated by this methylation site increased to 64.3% (Fig. 5).

**Table 3:** Mediating effect analysis of DNA methylation on difficulty falling asleep and female infertility

DNA ethylation site	Interaction effect	Total effect	Direct effect	Mediation effect (95%*CI*)	Mediation effect percentage
**Model 1**					
cg24524451	2.83	0.26^*^	0.19	0.07 (-0.02, 0.17)	24.0%
cg08298632	12.04	0.24	0.14	0.10 (0.01, 0.21)^*^	37.3%
cg16875032	-0.01	0.26^*^	0.20	0.06 (-0.01, 0.14)	19.1%
cg01166932	5.18	0.26^*^	0.21	0.05 (-0.03, 0.15)	19.0%
cg21700663	-2.42	0.26^*^	0.20	0.06 (-0.01, 0.14)	19.7%
cg22915785	6.81	0.25^*^	0.18	0.07 (-0.02, 0.16)	25.3%
cg03029255	6.26	0.24^*^	0.18	0.06 (-0.01, 0.16)	23.8%
cg07770222	8.18	0.26^*^	0.19	0.07 (-0.01, 0.16)	24.5%
**Model 2**					
cg24524451	1.55	0.08	0.04	0.04 (-0.03, 0.13)	24.6%
cg08298632	22.08^*^	0.14	0.04	0.10 (0.01, 0.22)^*^	64.3%
cg16875032	-0.31	0.12	0.08	0.04 (-0.03, 0.14)	20.0%
cg01166932	4.51	0.13	0.08	0.05 (-0.04, 0.16)	31.8%
cg21700663	-1.21	0.10	0.06	0.04 (-0.01, 0.13)	18.7%
cg22915785	11.90^*^	0.08	0.04	0.04 (-0.02, 0.13)	27.9%
cg03029255	12.18^*^	0.06	0.01	0.05 (-0.02, 0.14)	29.6%
cg07770222	13.81^**^	0.10	0.05	0.05 (-0.02, 0.14)	32.3%

Model 1: unadjusted for any covariates; Model 2: adjusted for overweight and obesity rates, age at first sexual activity

^*^P < 0.05, ^**^P < 0.01

## Discussion

Infertility is a significant public health concern, and it is necessary to identify factors that contribute to infertility and tailor effective prevention interventions. To the best of our knowledge, this study is the first to report altered DNA methylation patterns in the whole blood of infertile women. We identified a total of 262 differential DNA methylation sites, with 180 sites showing significant hypermethylation and 82 sites showing hypomethylation in women with ovulation disorders compared to the normal fertility control group. The study revealed that difficulty falling asleep is associated with an increased risk of infertility in women of childbearing age. The causal mediation analysis identified specific DNA methylation sites (cg08298632) that mediate the relationship between difficulty falling asleep and infertility.

We found that difficulty falling asleep was a risk factor for infertility in women. This is consistent with previous studies. A study conducted on 1,176 couples in North America also reported reduced fertility in couples where women experienced difficulty falling asleep compared to those with normal sleep patterns [18]. Another study conducted at Tangshan Maternal and Child Health Hospital found that poor sleep quality, late sleeping time, and insufficient sleep were all associated with an increased risk of infertility [35]. Previous studies have also shown that circadian disturbances affect various aspects of reproductive function, such as follicular development, hormone secretion, and regulation of the menstrual cycle; they have also been associated with an increased risk of spontaneous abortion and preterm birth [14, 36]. The sleep characteristics of women with infertility may vary depending on the underlying etiology, and studies have found that compared to women with unexplained infertility, women with PCOS report more daytime sleepiness [37, 38]. However, in the present study, daytime sleepiness was not found to be a risk factor for female infertility, despite its association with difficulty falling asleep. The most common sleep issues are difficulty falling asleep and excessive daytime sleepiness [39], difficulty in falling asleep commonly leads to daytime sleepiness, lethargy, and a general feeling of being unwell [40]. However, difficulty falling asleep and excessive daytime sleepiness are two different sleep issues with distinct symptoms, causes, and mechanisms [41]. Further research and in-depth analysis are necessary to explore these relationships and gain a deeper understanding of the mechanisms and factors influencing sleep and its impact on female infertility.

Studies investigating the mediating role of DNA methylation in the relationship between sleep and infertility are relatively limited. However, previous research has demonstrated that DNA methylation could potentially function as an intermediary mechanism linking environmental exposure and the onset of diseases, thereby influencing the relationship between these two factors through the regulation of gene expression [42, 43]. A prospective cohort study showed the mediating effect of DNA methylation in the association between maternal sleep during pregnancy and offspring adiposity status. Specific DNA methylation sites, such as cg04351668, cg12225226, and cg12232388 were found to significantly mediate the relationship between sleep midpoint and offspring’s subcutaneous fat and BMI [44]. Additionally, a survey outcome revealed that sperm DNA methylation mediates the association between male age and reproductive outcomes among couples undergoing infertility treatment [45].

In this study, the causal mediation analysis revealed that a DNA methylation site (cg08298632) played a positive mediating role in the relationship between difficulty falling asleep and infertility in women. This site is located on the *KCNC2* gene, which is a protein-coding gene that encodes components of voltage-gated potassium channels that regulate voltage-dependent potassium ion permeability in excitable membranes and is mainly expressed in the brain. Studies have shown that overexpression of the *KCNC2* gene in hippocampal neurons may lead to higher amplitude and faster dynamics of potassium currents, which are linked to neuronal hyperexcitability in patients with bipolar disorder [46]. Other studies have shown that *Kcnc1* is closely related to circadian rhythm. Knockout mice lacking *Kcnc1* and *Kcnc2* are unable to express Kv 3.1 and Kv 3.2 potassium channels in the suprachiasmatic nucleus, and the fast delayed rectifying potassium channel current is greatly reduced, showing an extremely disordered daily rhythm [47]. Furthermore, the *Kcnc2* polymorphism is also associated with reproductive traits in animals, such as litter size in sows [48]. In conclusion, we suggest that difficulty falling asleep among women may influence the occurrence of infertility through DNA methylation of the *KCNC2* gene. However, further research is needed to fully understand the underlying mechanisms and pathways involved in this association.

To the best of our knowledge, this study is the first to utilize patients with anovulatory infertility as the case group, while normal fertility females were used as the control group. The use of a DNA methylation 850 K chip for genome-wide DNA methylation analysis allowed for a comprehensive examination of the association between DNA methylation and infertility, shedding light on the epigenetic pathogenesis and mechanisms underlying female infertility. Causal mediation analysis was used to further explore the mediation effect of DNA methylation in the relationship between sleep quality and infertility in women, providing more robust evidence for the underlying biological mechanisms linking sleep quality and infertility, and providing a theoretical basis to develop strategies for the prevention and treatment of infertility.

This study has some limitations that should be considered. First, the assessment of sleep quality relied on self-report measures, which might lead to self-reporting bias. Second, due to the limited funding, the sample size of this study was small, and no comparative analysis of different types and causes of infertility was conducted. Increasing the sample size and conducting in-depth stratification analysis in future studies would provide more robust and comprehensive findings. Third, this study did not include in-depth functional studies of differentially methylated sites, methylated genes, and the selected genes with mediation effects identified from the methylated 850 K chips. Finally, while the causal mediation analysis provides valuable insights, it only suggests possible causal associations and cannot fully confirm the causal associations between difficulty falling asleep, DNA methylation, and infertility, which should be further verified by experimental studies in the future.

## Conclusion

In conclusion, the findings of this study suggest that DNA methylation sites play a significant role in mediating the relationship between difficulty falling asleep and infertility in women. Specifically, the DNA methylation site cg08298632 was found to be a mediating variable. These findings contribute to our understanding of the underlying mechanisms that connect difficulty falling asleep and infertility in women. Further studies are necessary to fully understand the biological significance and potential therapeutic applications of these findings. The identified DNA methylation sites provide new and valuable insights and potential targets for future studies aiming to prevent and treat female infertility.

### Supplementary Information

**Additional file 1: Table S1.** The details of the Reproductive Health of Childbearing Couples—Anhui Cohort (RHCC-AC). **Table S2.** Comparison of differential methylation sites between case and control group.

## Data Availability

The datasets generated and/or analysed during the current study are not publicly available but are available from the corresponding author on reasonable request.
