# Supplementary material for: Mediating effects of DNA methylation in the association between sleep quality and infertility among women of childbearing age
Source: BMC Public Health. 2023 Sep 15;23:1802. doi: 10.1186/s12889-023-16681-w (PMC10503064; doi:10.1186/s12889-023-16681-w)
Supplement: Supplementary file 1 — Additional file 1: Table S1. The details of the Reproductive Health of Childbearing Couples—Anhui Cohort (RHCC-AC). Table S2. Comparison of differential methylation sites between case and control group. [file 12889_2023_16681_MOESM1_ESM.docx]

Supplementary Material

| **Table S1. The details of the Reproductive Health of Childbearing Couples—Anhui Cohort (RHCC-AC)** | | | | | |
| --- | --- | --- | --- | --- | --- |
| **Subcohorts** | **Recruiting objects** | **Recruiting time** | **Recruiting locations** | **Inclusion criteria** | **Exclusion criteria** |
| Newlyweds cohort | Newlywed couples | 2019.4 - 2021.6 | Maternal and Child Health Care Family Planning Service Center in 16 cities/counties in Anhui Province, China | (1) Couples with informed consent;  (2) Couples who have never had children together;  (3) Couples who can understand and complete the questionnaires independently. | (1) Couples with serious organic disease or mental disease;  (2) Couples who show poor compliance, refusing to complete the questionnaires or unwilling to accept follow-up investigations. |
| Infertility-specific cohort | Infertile couples | 2020.12 - | Ma’anshan Maternal and Child Health Hospital, 901 Hospital of People’s Liberation Army | (1) Couples who had not conceived within 12 months after stopping the use of contraception;  (2) Couples with informed consent;  (3) Couples who can understand and complete the questionnaires independently. | (1) Couples with serious organic disease or mental disease;  (2) Couples who show poor compliance, refusing to complete the questionnaires or unwilling to accept follow-up investigations. |

| **Table S2.** Comparison of differential methylation sites between case and control group | | | | | | | | | | |
| --- | --- | --- | --- | --- | --- | --- | --- | --- | --- | --- |
| CpG sites | Cases Mean β value | Controls Mean β value | *△β* value | *P* value | BH.adjust *P* value | Chr | UCSC RefGene Name | Relation to UCSC CpG Island | UCSC RefGene Group | Type |
| cg05578102 | 0.46101069 | 0.347335337 | 0.113675353 | 0.003023205 | 12 | 0.049004567 | LOC100049716 | TSS200 | opensea | Hypermethylated |
| cg19665696 | 0.322306723 | 0.184248928 | 0.138057795 | 0.003013466 | 7 | 0.048905883 | ADAP1 | Body | Island | Hypermethylated |
| cg13638229 | 0.662944659 | 0.612255838 | 0.050688821 | 0.002952028 | 10 | 0.048260917 | EBF3 | Body | Island | Hypermethylated |
| cg04734610 | 0.609998578 | 0.535580693 | 0.074417885 | 0.002924067 | 4 | 0.047994183 |  | IGR | opensea | Hypermethylated |
| cg18120979 | 0.440066466 | 0.389435221 | 0.050631245 | 0.002913947 | 6 | 0.047883558 | DUSP22 | Body | opensea | Hypermethylated |
| cg25610945 | 0.822672102 | 0.734584824 | 0.088087278 | 0.002889597 | 1 | 0.047621509 |  | IGR | opensea | Hypermethylated |
| cg17212350 | 0.257113962 | 0.199441512 | 0.05767245 | 0.002721969 | 7 | 0.045948412 | ABCB4 | Body | opensea | Hypermethylated |
| cg18668511 | 0.711450563 | 0.623442725 | 0.088007838 | 0.002682238 | 5 | 0.045515759 | PCDHB8 | TSS1500 | N_Shore | Hypermethylated |
| cg00088688 | 0.660832312 | 0.609430272 | 0.05140204 | 0.002538868 | 6 | 0.044006664 |  | IGR | opensea | Hypermethylated |
| cg20192814 | 0.813039606 | 0.739267423 | 0.073772183 | 0.002534293 | 13 | 0.04396289 |  | IGR | opensea | Hypermethylated |
| cg26020695 | 0.776323628 | 0.711898257 | 0.064425371 | 0.002460058 | 13 | 0.043175575 | SIAH3 | 3'UTR | opensea | Hypermethylated |
| cg05185784 | 0.404957736 | 0.353166059 | 0.051791678 | 0.002402573 | 16 | 0.04254442 | DEF8 | 5'UTR | Island | Hypermethylated |
| cg01201512 | 0.58858518 | 0.371830938 | 0.216754243 | 0.00235604 | 12 | 0.042053974 | NINJ2 | Body | opensea | Hypermethylated |
| cg03082247 | 0.824010068 | 0.762807303 | 0.061202765 | 0.002341682 | 8 | 0.041906378 |  | IGR | opensea | Hypermethylated |
| cg04664516 | 0.41097364 | 0.336698888 | 0.074274752 | 0.002338326 | 10 | 0.041875442 | PTEN | 5'UTR | opensea | Hypermethylated |
| cg09470311 | 0.626338825 | 0.559043037 | 0.067295788 | 0.002312129 | 12 | 0.041610313 | MYRFL | Body | opensea | Hypermethylated |
| cg07770222 | 0.642564817 | 0.568712556 | 0.073852261 | 0.002293008 | 8 | 0.04140935 | C8orf31 | TSS1500 | opensea | Hypermethylated |
| cg24000535 | 0.552092856 | 0.499447267 | 0.052645588 | 0.002228803 | 14 | 0.040691812 | LOC101928909 | Body | opensea | Hypermethylated |
| cg24797210 | 0.908778111 | 0.835434807 | 0.073343305 | 0.002200618 | 10 | 0.040362248 | SVIL | Body | opensea | Hypermethylated |
| cg09372928 | 0.281120921 | 0.340035652 | -0.058914731 | 0.002153281 | 1 | 0.039852195 | CAMTA1 | 1stExon | Island | Hypomethylated |
| cg18888461 | 0.451210197 | 0.397967273 | 0.053242924 | 0.002139594 | 11 | 0.039673623 | LDHAL6A | 1stExon | Island | Hypermethylated |
| cg12159023 | 0.715598963 | 0.657687546 | 0.057911417 | 0.002035315 | 19 | 0.038480911 | HCN2 | Body | Island | Hypermethylated |
| cg12060786 | 0.286675396 | 0.16562752 | 0.121047875 | 0.001998903 | 6 | 0.038065027 | C6orf48 | 5'UTR | S_Shore | Hypermethylated |
| cg24391982 | 0.419502196 | 0.475426073 | -0.055923877 | 0.001967244 | 6 | 0.037695975 | THBS2 | Body | N_Shore | Hypomethylated |
| cg10617577 | 0.700019381 | 0.620253451 | 0.07976593 | 0.001939099 | 10 | 0.037372464 |  | IGR | opensea | Hypermethylated |
| cg02249088 | 0.37604741 | 0.322235679 | 0.053811731 | 0.001921761 | 11 | 0.037164683 |  | IGR | opensea | Hypermethylated |
| cg03486986 | 0.275887677 | 0.22321485 | 0.052672827 | 0.001873333 | 13 | 0.036548562 |  | IGR | S_Shore | Hypermethylated |
| cg03456771 | 0.63408573 | 0.693578309 | -0.05949258 | 0.001595694 | 7 | 0.033049372 |  | IGR | N_Shore | Hypomethylated |
| cg08029281 | 0.343582314 | 0.400251587 | -0.056669273 | 0.001528898 | 1 | 0.032174755 |  | IGR | Island | Hypomethylated |
| cg21523688 | 0.310494726 | 0.363622793 | -0.053128068 | 0.001501551 | 15 | 0.031809545 | SORD | Body | S_Shelf | Hypomethylated |
| cg03029255 | 0.671165475 | 0.604100344 | 0.067065131 | 0.001401924 | 8 | 0.030479529 | C8orf31 | 1stExon | opensea | Hypermethylated |
| cg07782112 | 0.699137287 | 0.594069722 | 0.105067564 | 0.001354882 | 2 | 0.0298081 |  | IGR | opensea | Hypermethylated |
| cg02380802 | 0.579280015 | 0.518763189 | 0.060516826 | 0.001328026 | 16 | 0.02941576 |  | IGR | S_Shore | Hypermethylated |
| cg26654770 | 0.440981868 | 0.27718694 | 0.163794928 | 0.001306283 | 12 | 0.029132982 | NINJ2 | Body | opensea | Hypermethylated |
| cg22716280 | 0.685273431 | 0.626481563 | 0.058791868 | 0.001249126 | 10 | 0.028343886 |  | IGR | N_Shelf | Hypermethylated |
| cg15994330 | 0.790549061 | 0.723209456 | 0.067339605 | 0.001216551 | 17 | 0.027893372 | TEX14 | 5'UTR | opensea | Hypermethylated |
| cg01798352 | 0.565478814 | 0.511932194 | 0.05354662 | 0.00110045 | 6 | 0.026156073 | PACRG | Body | opensea | Hypermethylated |
| cg03105319 | 0.702143029 | 0.75833214 | -0.056189111 | 0.00104227 | 3 | 0.025283837 | FHIT | 5'UTR | opensea | Hypomethylated |
| cg13808979 | 0.496775113 | 0.433430846 | 0.063344267 | 0.001022449 | 6 | 0.024967442 | PSORS1C1 | 5'UTR | opensea | Hypermethylated |
| cg08540654 | 0.60728255 | 0.542160245 | 0.065122305 | 0.001007226 | 8 | 0.024730125 | C8orf31 | TSS200 | opensea | Hypermethylated |
| cg22915785 | 0.637098469 | 0.560902145 | 0.076196323 | 0.000976663 | 8 | 0.024238224 | C8orf31 | TSS1500 | opensea | Hypermethylated |
| cg23180489 | 0.664530912 | 0.613829612 | 0.0507013 | 0.000974995 | 8 | 0.02421149 | LYNX1 | TSS200 | S_Shore | Hypermethylated |
| cg05028929 | 0.612787905 | 0.528348545 | 0.084439359 | 0.00095844 | 16 | 0.02395189 | C1QTNF8 | 3'UTR | N_Shore | Hypermethylated |
| cg05375686 | 0.641279018 | 0.587282473 | 0.053996545 | 0.000914642 | 12 | 0.023257638 |  | IGR | Island | Hypermethylated |
| cg16553796 | 0.34921305 | 0.401167546 | -0.051954496 | 0.000860783 | 18 | 0.02236976 | PARD6G | TSS200 | S_Shore | Hypomethylated |
| cg14506366 | 0.810761761 | 0.861412217 | -0.050650456 | 0.000774535 | 5 | 0.02092482 | SLC6A3 | Body | opensea | Hypomethylated |
| cg13800828 | 0.766707499 | 0.712954757 | 0.053752743 | 0.00076543 | 12 | 0.020760279 |  | IGR | N_Shelf | Hypermethylated |
| cg22048216 | 0.294479926 | 0.35618299 | -0.061703064 | 0.000707431 | 18 | 0.019724569 |  | IGR | Island | Hypomethylated |
| cg03147852 | 0.658371515 | 0.70901027 | -0.050638755 | 0.000683096 | 16 | 0.019300246 | GAN | Body | S_Shelf | Hypomethylated |
| cg21700663 | 0.65771143 | 0.548967825 | 0.108743604 | 0.000655276 | 15 | 0.018807043 |  | IGR | N_Shore | Hypermethylated |
| cg01166932 | 0.476263753 | 0.401082881 | 0.075180872 | 0.000652156 | 15 | 0.018747423 | CGNL1 | 5'UTR | opensea | Hypermethylated |
| cg20195067 | 0.597244435 | 0.529818401 | 0.067426034 | 0.000635029 | 17 | 0.01843348 | TEX14 | 5'UTR | opensea | Hypermethylated |
| cg13428516 | 0.231099486 | 0.283602722 | -0.052503236 | 0.000568915 | 19 | 0.017209191 | MAMSTR | TSS1500 | Island | Hypomethylated |
| cg02541142 | 0.683650683 | 0.611690277 | 0.071960405 | 0.000552436 | 12 | 0.016878518 | MYRFL | Body | opensea | Hypermethylated |
| cg10663765 | 0.582888854 | 0.639918917 | -0.057030063 | 0.000543735 | 3 | 0.016714005 |  | IGR | Island | Hypomethylated |
| cg16102063 | 0.354281055 | 0.40534096 | -0.051059905 | 0.000494676 | 8 | 0.015720547 | PPP3CC | TSS1500 | Island | Hypomethylated |
| cg23971638 | 0.459380068 | 0.384536666 | 0.074843403 | 0.000458153 | 12 | 0.014979387 | NINJ2 | Body | opensea | Hypermethylated |
| cg19084031 | 0.257323095 | 0.145244564 | 0.112078532 | 0.000448087 | 15 | 0.014767587 |  | IGR | N_Shelf | Hypermethylated |
| cg00399059 | 0.648336014 | 0.585025278 | 0.063310736 | 0.000368989 | 8 | 0.013073335 | LYNX1 | TSS200 | S_Shore | Hypermethylated |
| cg11569930 | 0.51884063 | 0.4677559 | 0.051084729 | 0.000359603 | 1 | 0.012880487 | DPM3 | TSS1500 | S_Shore | Hypermethylated |
| cg03455137 | 0.617913811 | 0.566254791 | 0.051659019 | 0.000356245 | 11 | 0.012805655 |  | IGR | opensea | Hypermethylated |
| cg12360753 | 0.412884238 | 0.359091398 | 0.053792839 | 0.000353305 | 4 | 0.012733502 |  | IGR | opensea | Hypermethylated |
| cg16875032 | 0.25409827 | 0.32895102 | -0.07485275 | 0.000340512 | 8 | 0.01244593 | SNTB1 | 1stExon | Island | Hypomethylated |
| cg19423170 | 0.40297753 | 0.351938022 | 0.051039508 | 0.000329497 | 21 | 0.012193391 | APP | Body | opensea | Hypermethylated |
| cg08709360 | 0.862060676 | 0.810370364 | 0.051690312 | 0.000296927 | 1 | 0.011399181 |  | IGR | Island | Hypermethylated |
| cg20104018 | 0.825970898 | 0.877652946 | -0.051682047 | 0.000296338 | 19 | 0.011387846 | C19orf61 | 3'UTR | opensea | Hypomethylated |
| cg17858911 | 0.422800377 | 0.499468404 | -0.076668027 | 0.000295544 | 12 | 0.011371561 | RSRC2 | TSS1500 | S_Shore | Hypomethylated |
| cg01244934 | 0.845428475 | 0.7879389 | 0.057489575 | 0.000273386 | 1 | 0.010809327 |  | IGR | Island | Hypermethylated |
| cg26878995 | 0.43212542 | 0.48345468 | -0.051329261 | 0.000267671 | 1 | 0.010655824 | GPR161 | TSS1500 | S_Shore | Hypomethylated |
| cg23322479 | 0.709801429 | 0.470036135 | 0.239765293 | 0.000260523 | 4 | 0.010477687 |  | IGR | opensea | Hypermethylated |
| cg00061868 | 0.605803318 | 0.550164576 | 0.055638741 | 0.000233563 | 13 | 0.009766075 | CYSLTR2 | 5'UTR | opensea | Hypermethylated |
| cg21570209 | 0.445059623 | 0.506750706 | -0.061691083 | 0.00023028 | 19 | 0.009672023 | FOXA3 | Body | S_Shore | Hypomethylated |
| cg26880777 | 0.453322618 | 0.402497297 | 0.05082532 | 0.000228601 | 1 | 0.009626338 | MUL1 | TSS1500 | S_Shore | Hypermethylated |
| cg19547155 | 0.439944592 | 0.374737328 | 0.065207264 | 0.000221034 | 19 | 0.009412069 | LAIR2 | TSS200 | opensea | Hypermethylated |
| cg12068625 | 0.668458328 | 0.608108729 | 0.060349599 | 0.000206745 | 7 | 0.009010865 | PDE1C | Body | opensea | Hypermethylated |
| cg09692889 | 0.869867454 | 0.924222093 | -0.054354639 | 0.000199802 | 1 | 0.008795153 | SMYD3 | Body | opensea | Hypomethylated |
| cg22497879 | 0.320253363 | 0.375578634 | -0.05532527 | 0.000195012 | 2 | 0.008662467 | GLS | TSS200 | Island | Hypomethylated |
| cg12084908 | 0.375937241 | 0.312024545 | 0.063912696 | 0.000193665 | 10 | 0.008626059 | SLC16A9 | Body | opensea | Hypermethylated |
| cg23492789 | 0.602616882 | 0.535390338 | 0.067226544 | 0.000192849 | 2 | 0.008602692 | EXOC6B | Body | opensea | Hypermethylated |
| cg06636551 | 0.328727757 | 0.248181325 | 0.080546432 | 0.00018886 | 8 | 0.008489503 | SPAG1 | Body | N_Shore | Hypermethylated |
| cg11824509 | 0.517983366 | 0.451802294 | 0.066181073 | 0.000185353 | 10 | 0.008391764 | C10orf57 | TSS1500 | N_Shore | Hypermethylated |
| cg26258108 | 0.199107952 | 0.284908782 | -0.085800829 | 0.000178687 | 2 | 0.008203404 | NCL | 1stExon | Island | Hypomethylated |
| cg17218799 | 0.649937199 | 0.598653312 | 0.051283887 | 0.00017808 | 14 | 0.008187604 | UNC79 | Body | opensea | Hypermethylated |
| cg07418777 | 0.633284685 | 0.580267524 | 0.053017161 | 0.000178073 | 10 | 0.008187604 | CCDC3 | Body | opensea | Hypermethylated |
| cg10271495 | 0.767974988 | 0.708908481 | 0.059066507 | 0.000171556 | 3 | 0.008007749 |  | IGR | opensea | Hypermethylated |
| cg08298632 | 0.434578207 | 0.373242157 | 0.06133605 | 0.000168776 | 12 | 0.007922164 | KCNC2 | Body | opensea | Hypermethylated |
| cg22891371 | 0.619667629 | 0.563059791 | 0.056607838 | 0.000163824 | 1 | 0.00777713 |  | IGR | N_Shelf | Hypermethylated |
| cg13135812 | 0.494448298 | 0.444048863 | 0.050399435 | 0.000160114 | 16 | 0.007666013 | LOC102724084 | Body | opensea | Hypermethylated |
| cg25410233 | 0.592688701 | 0.699932713 | -0.107244012 | 0.000151371 | 1 | 0.007409695 | SLC35F3 | Body | opensea | Hypomethylated |
| cg00800759 | 0.392637309 | 0.319479458 | 0.073157851 | 0.000150747 | 16 | 0.007389424 | CRYM | 5'UTR | N_Shelf | Hypermethylated |
| cg00016814 | 0.82403292 | 0.886660326 | -0.062627405 | 0.0001489 | 7 | 0.007335666 | CNTNAP2 | Body | opensea | Hypomethylated |
| cg04736217 | 0.500341843 | 0.42171418 | 0.078627663 | 0.000146638 | 6 | 0.007269766 | C6orf48 | 5'UTR | S_Shore | Hypermethylated |
| cg08474748 | 0.48928098 | 0.545724454 | -0.056443474 | 0.00014641 | 5 | 0.007264395 |  | IGR | Island | Hypomethylated |
| cg20834311 | 0.528455267 | 0.478093379 | 0.050361888 | 0.00014271 | 2 | 0.007152915 |  | IGR | opensea | Hypermethylated |
| cg08644860 | 0.454398219 | 0.400473667 | 0.053924552 | 0.000142091 | 14 | 0.007133619 | CEP128 | Body | opensea | Hypermethylated |
| cg07526328 | 0.467945348 | 0.399833532 | 0.068111816 | 0.000141826 | 18 | 0.007125876 | IMPACT | Body | S_Shelf | Hypermethylated |
| cg09087363 | 0.735376939 | 0.789953214 | -0.054576275 | 0.000134322 | 16 | 0.006878562 |  | IGR | Island | Hypomethylated |
| cg20638675 | 0.307966404 | 0.360646129 | -0.052679725 | 0.000133932 | 10 | 0.006868153 |  | IGR | opensea | Hypomethylated |
| cg26894311 | 0.382984384 | 0.437584261 | -0.054599877 | 0.000122049 | 10 | 0.006482572 | NFKB2 | TSS1500 | Island | Hypomethylated |
| cg11104311 | 0.523283255 | 0.653723802 | -0.130440547 | 0.000118277 | 1 | 0.006364342 | SLC35F3 | Body | opensea | Hypomethylated |
| cg09700085 | 0.37173006 | 0.423066016 | -0.051335956 | 0.000115842 | 3 | 0.006284731 | SLC6A20 | 1stExon | Island | Hypomethylated |
| cg08679807 | 0.583618532 | 0.533173031 | 0.050445501 | 0.000113621 | 1 | 0.006214318 | ACOT7 | Body | N_Shore | Hypermethylated |
| cg07806915 | 0.746896729 | 0.690704265 | 0.056192464 | 0.000100809 | 18 | 0.005757828 | CTIF | Body | opensea | Hypermethylated |
| cg27543672 | 0.832605199 | 0.885787727 | -0.053182529 | 9.84056E-05 | 1 | 0.005669426 |  | IGR | N_Shelf | Hypomethylated |
| cg01242309 | 0.737685569 | 0.68636917 | 0.051316399 | 9.06077E-05 | 20 | 0.005380037 |  | IGR | N_Shelf | Hypermethylated |
| cg12902040 | 0.742707565 | 0.681220669 | 0.061486896 | 8.98446E-05 | 18 | 0.00535429 |  | IGR | N_Shelf | Hypermethylated |
| cg00145284 | 0.568085472 | 0.504513422 | 0.063572049 | 8.88518E-05 | 1 | 0.005313891 |  | IGR | opensea | Hypermethylated |
| cg04754212 | 0.513178278 | 0.46241766 | 0.050760617 | 8.82797E-05 | 10 | 0.005296444 | UROS | 5'UTR | opensea | Hypermethylated |
| cg14041338 | 0.467269121 | 0.410872416 | 0.056396704 | 8.17042E-05 | 13 | 0.005053801 | ENOX1 | Body | opensea | Hypermethylated |
| cg23627062 | 0.779751847 | 0.831464173 | -0.051712326 | 8.07363E-05 | 2 | 0.005017098 | LOC285045 | TSS200 | opensea | Hypomethylated |
| cg25390025 | 0.770243028 | 0.822376694 | -0.052133667 | 8.05793E-05 | 17 | 0.005010721 | APOH | TSS200 | opensea | Hypomethylated |
| cg04657419 | 0.646958784 | 0.592502927 | 0.054455857 | 7.56466E-05 | 5 | 0.004816601 | C5orf43 | 3'UTR | N_Shelf | Hypermethylated |
| cg17810781 | 0.685506529 | 0.632163845 | 0.053342684 | 7.54174E-05 | 1 | 0.004809069 | CACNA1S | TSS1500 | N_Shore | Hypermethylated |
| cg21046659 | 0.199189367 | 0.142630106 | 0.05655926 | 7.42822E-05 | 9 | 0.004762649 | SUSD1 | 1stExon | Island | Hypermethylated |
| cg03471195 | 0.638077785 | 0.567711678 | 0.070366107 | 7.38272E-05 | 12 | 0.004741692 | NINJ2 | Body | opensea | Hypermethylated |
| cg24945462 | 0.36648297 | 0.42415406 | -0.057671089 | 7.30695E-05 | 21 | 0.004714404 | LOC642852 | Body | Island | Hypomethylated |
| cg09111438 | 0.535562886 | 0.482855597 | 0.052707289 | 7.13145E-05 | 12 | 0.004651421 |  | IGR | opensea | Hypermethylated |
| cg05972386 | 0.792399713 | 0.741635817 | 0.050763896 | 6.9706E-05 | 2 | 0.004579576 | GTF2A1L | TSS200 | Island | Hypermethylated |
| cg08872579 | 0.572475277 | 0.499463674 | 0.073011602 | 6.81952E-05 | 1 | 0.004526805 | TTC22 | Body | N_Shore | Hypermethylated |
| cg15822108 | 0.630749571 | 0.571157065 | 0.059592506 | 6.66601E-05 | 4 | 0.004466351 | NAA15 | Body | opensea | Hypermethylated |
| cg27528727 | 0.58665931 | 0.522624202 | 0.064035108 | 6.5412E-05 | 17 | 0.004416064 | PPM1E | Body | opensea | Hypermethylated |
| cg01586844 | 0.566030939 | 0.506781889 | 0.059249051 | 6.46672E-05 | 19 | 0.004383052 | SIPA1L3 | 5'UTR | opensea | Hypermethylated |
| cg14752361 | 0.732852427 | 0.7962399 | -0.063387473 | 6.44763E-05 | 7 | 0.004375343 | MACC1 | 5'UTR | opensea | Hypomethylated |
| cg00047185 | 0.600955656 | 0.52153355 | 0.079422107 | 6.29036E-05 | 19 | 0.00431472 | MIR526B | TSS1500 | opensea | Hypermethylated |
| cg25758263 | 0.624236384 | 0.552806622 | 0.071429763 | 6.06537E-05 | 5 | 0.004224103 | TMEM161B | Body | opensea | Hypermethylated |
| cg12152193 | 0.503106656 | 0.445729445 | 0.057377211 | 5.47787E-05 | 5 | 0.003973066 | RNF44 | 3'UTR | opensea | Hypermethylated |
| cg07017074 | 0.624148314 | 0.572399678 | 0.051748635 | 5.41244E-05 | 1 | 0.003943078 | KIAA1324 | Body | opensea | Hypermethylated |
| cg02832476 | 0.340676963 | 0.28187848 | 0.058798483 | 5.3593E-05 | 2 | 0.003919714 |  | IGR | opensea | Hypermethylated |
| cg04147657 | 0.739484605 | 0.674280142 | 0.065204463 | 5.22139E-05 | 18 | 0.003861163 | C18orf32 | 3'UTR | opensea | Hypermethylated |
| cg11050198 | 0.365303373 | 0.419284203 | -0.05398083 | 4.93023E-05 | 9 | 0.003731245 | TMEM8B | TSS1500 | Island | Hypomethylated |
| cg14178013 | 0.723730352 | 0.774236354 | -0.050506002 | 4.9182E-05 | 9 | 0.003726726 | PALM2-AKAP2 | Body | opensea | Hypomethylated |
| cg11853729 | 0.532123965 | 0.480362975 | 0.05176099 | 4.74418E-05 | 6 | 0.003649292 | NUDT3 | Body | opensea | Hypermethylated |
| cg13766911 | 0.382241041 | 0.322129586 | 0.060111454 | 4.70795E-05 | 16 | 0.00362848 | FUS | ExonBnd | opensea | Hypermethylated |
| cg00191853 | 0.449057169 | 0.348702839 | 0.10035433 | 4.67707E-05 | 8 | 0.003618442 | SPAG1 | Body | opensea | Hypermethylated |
| cg26508775 | 0.666178299 | 0.600975795 | 0.065202504 | 4.59379E-05 | 20 | 0.003579956 | RTFDC1 | Body | opensea | Hypermethylated |
| cg14269096 | 0.639650856 | 0.581759836 | 0.057891019 | 4.56521E-05 | 18 | 0.003568198 | ZNF532 | 5'UTR | S_Shore | Hypermethylated |
| cg07661849 | 0.475027602 | 0.421920612 | 0.05310699 | 4.56082E-05 | 4 | 0.003565955 | ZNF827 | Body | S_Shelf | Hypermethylated |
| cg24524451 | 0.367566666 | 0.43908542 | -0.071518753 | 4.44121E-05 | 7 | 0.003510812 | ADCK2 | TSS1500 | N_Shore | Hypomethylated |
| cg22861548 | 0.634813649 | 0.559464585 | 0.075349064 | 4.32302E-05 | 8 | 0.003455941 | LYNX1 | TSS200 | S_Shore | Hypermethylated |
| cg11186468 | 0.71117199 | 0.65057147 | 0.06060052 | 4.22257E-05 | 19 | 0.003416045 | HAUS5 | ExonBnd | opensea | Hypermethylated |
| cg06751596 | 0.18579115 | 0.129923235 | 0.055867915 | 4.12003E-05 | 5 | 0.003373094 | SKP1 | TSS200 | Island | Hypermethylated |
| cg07937578 | 0.442914057 | 0.384893627 | 0.05802043 | 4.09129E-05 | 4 | 0.003359641 |  | IGR | opensea | Hypermethylated |
| cg20272813 | 0.496451117 | 0.442128436 | 0.054322681 | 3.99088E-05 | 20 | 0.003304442 | PLCG1 | TSS1500 | N_Shore | Hypermethylated |
| cg12533206 | 0.671852831 | 0.619304328 | 0.052548503 | 3.92542E-05 | 10 | 0.003273987 | ADARB2 | Body | opensea | Hypermethylated |
| cg08364334 | 0.828148009 | 0.774119473 | 0.054028536 | 3.876E-05 | 4 | 0.003252458 |  | IGR | N_Shelf | Hypermethylated |
| cg24956366 | 0.693785965 | 0.640583196 | 0.053202769 | 3.85958E-05 | 3 | 0.003243851 | UBE2E2 | Body | opensea | Hypermethylated |
| cg02869243 | 0.486237896 | 0.430376502 | 0.055861393 | 3.74819E-05 | 14 | 0.003190087 | SERPINA12 | 5'UTR | opensea | Hypermethylated |
| cg13311549 | 0.765817824 | 0.824180569 | -0.058362745 | 3.73493E-05 | 7 | 0.003184863 | OSBPL3 | 5'UTR | opensea | Hypomethylated |
| cg20325636 | 0.611394775 | 0.554103668 | 0.057291108 | 3.46868E-05 | 18 | 0.003059088 | ZNF519 | Body | opensea | Hypermethylated |
| cg01899581 | 0.707029378 | 0.654814814 | 0.052214564 | 3.40096E-05 | 13 | 0.003019968 | DIAPH3 | Body | N_Shelf | Hypermethylated |
| cg04282723 | 0.66537347 | 0.609472095 | 0.055901375 | 3.3793E-05 | 11 | 0.00300726 | AP2A2 | Body | Island | Hypermethylated |
| cg16435426 | 0.645442756 | 0.593544022 | 0.051898734 | 3.29197E-05 | 2 | 0.002963191 |  | IGR | opensea | Hypermethylated |
| cg18640030 | 0.625028403 | 0.569731539 | 0.055296865 | 3.26581E-05 | 8 | 0.002948649 | CRH | TSS200 | S_Shore | Hypermethylated |
| cg27264311 | 0.625130622 | 0.57115832 | 0.053972303 | 3.14529E-05 | 7 | 0.002887286 | GS1-124K5.11 | Body | opensea | Hypermethylated |
| cg19149114 | 0.525355575 | 0.435454502 | 0.089901073 | 2.99036E-05 | 2 | 0.002801557 |  | IGR | opensea | Hypermethylated |
| cg08718880 | 0.746973912 | 0.685752276 | 0.061221636 | 2.90493E-05 | 1 | 0.002756586 | TNNI3K | Body | opensea | Hypermethylated |
| cg01457413 | 0.247894145 | 0.29884371 | -0.050949565 | 2.86645E-05 | 19 | 0.002736289 | ZNF846 | TSS200 | Island | Hypomethylated |
| cg06601130 | 0.545996464 | 0.488665093 | 0.057331372 | 2.78033E-05 | 2 | 0.002688504 |  | IGR | S_Shelf | Hypermethylated |
| cg14783184 | 0.769809874 | 0.822137299 | -0.052327424 | 2.52598E-05 | 6 | 0.002551519 |  | IGR | opensea | Hypomethylated |
| cg06904349 | 0.640088451 | 0.569006374 | 0.071082077 | 2.50438E-05 | 1 | 0.002540069 | KCNAB2 | Body | opensea | Hypermethylated |
| cg01930417 | 0.241624655 | 0.152361808 | 0.089262847 | 2.44458E-05 | 11 | 0.002502853 |  | IGR | Island | Hypermethylated |
| cg11715789 | 0.290544344 | 0.340704895 | -0.050160552 | 2.40498E-05 | 5 | 0.002481622 | RUFY1 | Body | opensea | Hypomethylated |
| cg05984044 | 0.317100727 | 0.372750923 | -0.055650197 | 2.11822E-05 | 4 | 0.002296018 | GABRB1 | Body | N_Shore | Hypomethylated |
| cg05754259 | 0.491599701 | 0.414874299 | 0.076725402 | 2.0915E-05 | 6 | 0.002281589 |  | IGR | opensea | Hypermethylated |
| cg21423178 | 0.636685217 | 0.573761624 | 0.062923593 | 1.84085E-05 | 10 | 0.002131796 | ST8SIA6-AS1 | TSS1500 | opensea | Hypermethylated |
| cg01375437 | 0.54206755 | 0.48999782 | 0.05206973 | 1.70458E-05 | 9 | 0.002037074 | LINC01501 | Body | opensea | Hypermethylated |
| cg22837490 | 0.728850464 | 0.676117574 | 0.05273289 | 1.6886E-05 | 7 | 0.002027839 |  | IGR | N_Shore | Hypermethylated |
| cg01959412 | 0.323910493 | 0.380399251 | -0.056488758 | 1.6873E-05 | 4 | 0.00202702 | MSX1 | 5'UTR | Island | Hypomethylated |
| cg25172788 | 0.569021225 | 0.518535195 | 0.050486029 | 1.61059E-05 | 9 | 0.001975143 | RMI1 | 5'UTR | opensea | Hypermethylated |
| cg20721090 | 0.646549868 | 0.587048884 | 0.059500984 | 1.54348E-05 | 3 | 0.001927395 | MCCC1 | Body | opensea | Hypermethylated |
| cg17611291 | 0.651144209 | 0.592677887 | 0.058466322 | 1.53971E-05 | 19 | 0.0019253 | ZNF792 | Body | opensea | Hypermethylated |
| cg14256587 | 0.236977787 | 0.289054356 | -0.052076569 | 1.41937E-05 | 2 | 0.001839773 | SNTG2 | TSS1500 | Island | Hypomethylated |
| cg08201238 | 0.734732371 | 0.676926144 | 0.057806227 | 1.41364E-05 | 16 | 0.00183643 | RAB11FIP3 | Body | N_Shore | Hypermethylated |
| cg17733590 | 0.575405602 | 0.511056431 | 0.064349171 | 1.34161E-05 | 19 | 0.001787089 | ZNF146 | 5'UTR | S_Shelf | Hypermethylated |
| cg10143433 | 0.462577691 | 0.52636326 | -0.063785569 | 1.27677E-05 | 3 | 0.001740355 | KLHL24 | 5'UTR | Island | Hypomethylated |
| cg09915421 | 0.64588991 | 0.588305951 | 0.057583959 | 1.25353E-05 | 17 | 0.001721106 |  | IGR | S_Shelf | Hypermethylated |
| cg03457729 | 0.359741558 | 0.419091304 | -0.059349746 | 1.24237E-05 | 15 | 0.001714461 |  | IGR | N_Shore | Hypomethylated |
| cg21304211 | 0.236304371 | 0.286495952 | -0.05019158 | 1.20095E-05 | 1 | 0.001684699 | C1orf183 | Body | Island | Hypomethylated |
| cg10840412 | 0.319243699 | 0.373829808 | -0.054586109 | 1.15377E-05 | 1 | 0.001651539 | GNG4 | TSS200 | Island | Hypomethylated |
| cg05979549 | 0.619497852 | 0.557751333 | 0.06174652 | 1.15177E-05 | 1 | 0.001649629 |  | IGR | opensea | Hypermethylated |
| cg08811958 | 0.761611675 | 0.704421993 | 0.057189682 | 1.14435E-05 | 20 | 0.001644443 | WISP2 | Body | opensea | Hypermethylated |
| cg17044356 | 0.718845312 | 0.769879567 | -0.051034255 | 1.08739E-05 | 17 | 0.001604948 | TMEM88 | TSS200 | N_Shore | Hypomethylated |
| cg01226278 | 0.602192083 | 0.549237396 | 0.052954688 | 1.02136E-05 | 10 | 0.00155505 | RAB11FIP2 | Body | opensea | Hypermethylated |
| cg06274207 | 0.592866111 | 0.648787617 | -0.055921506 | 1.02014E-05 | 11 | 0.001553832 | LOC101928847 | Body | N_Shore | Hypomethylated |
| cg18150468 | 0.557406926 | 0.50521507 | 0.052191855 | 1.00451E-05 | 7 | 0.00154215 | GSAP | Body | opensea | Hypermethylated |
| cg12290615 | 0.412227198 | 0.350450873 | 0.061776325 | 9.98294E-06 | 6 | 0.001536797 | SMAP1 | Body | opensea | Hypermethylated |
| cg23496400 | 0.344229698 | 0.39948435 | -0.055254652 | 9.58657E-06 | 2 | 0.001504862 | VSNL1 | TSS200 | Island | Hypomethylated |
| cg24156331 | 0.780290676 | 0.832585078 | -0.052294402 | 9.51212E-06 | 14 | 0.001499526 | ITPK1 | Body | opensea | Hypomethylated |
| cg07869345 | 0.842350795 | 0.897336672 | -0.054985876 | 9.20751E-06 | 6 | 0.001470338 | ZFAND3 | Body | opensea | Hypomethylated |
| cg20852378 | 0.536614607 | 0.484676988 | 0.051937618 | 9.14573E-06 | 4 | 0.001466972 |  | IGR | S_Shore | Hypermethylated |
| cg09202227 | 0.24513488 | 0.297081313 | -0.051946433 | 9.07632E-06 | 16 | 0.001459727 | SMPD3 | TSS1500 | Island | Hypomethylated |
| cg26381322 | 0.283923332 | 0.343381804 | -0.059458472 | 7.6062E-06 | 1 | 0.001326101 | BATF3 | 1stExon | Island | Hypomethylated |
| cg14868223 | 0.591205784 | 0.535147339 | 0.056058445 | 6.74179E-06 | 19 | 0.001239281 |  | IGR | opensea | Hypermethylated |
| cg03210210 | 0.719197777 | 0.772990726 | -0.053792949 | 6.50098E-06 | 5 | 0.001214098 | TNFAIP8 | Body | N_Shore | Hypomethylated |
| cg03530210 | 0.240069963 | 0.291931077 | -0.051861114 | 6.49101E-06 | 1 | 0.00121295 | WDR47 | 1stExon | Island | Hypomethylated |
| cg18650263 | 0.502258554 | 0.437898878 | 0.064359676 | 6.17233E-06 | 6 | 0.001183007 | CCR6 | TSS1500 | opensea | Hypermethylated |
| cg16471084 | 0.242976039 | 0.295652633 | -0.052676595 | 6.04517E-06 | 6 | 0.001168363 | TMEM170B | TSS1500 | Island | Hypomethylated |
| cg05309750 | 0.553729969 | 0.499626174 | 0.054103794 | 5.6803E-06 | 3 | 0.001130444 | GXYLT2 | Body | opensea | Hypermethylated |
| cg04797274 | 0.568486988 | 0.507117202 | 0.061369787 | 5.65434E-06 | 1 | 0.001129093 | C8B | TSS1500 | opensea | Hypermethylated |
| cg17276652 | 0.762703272 | 0.700417937 | 0.062285335 | 5.58741E-06 | 1 | 0.001120147 | MSH4 | Body | opensea | Hypermethylated |
| cg08224569 | 0.40907763 | 0.473237964 | -0.064160334 | 5.04522E-06 | 8 | 0.001059298 | GPR20 | 5'UTR | S_Shore | Hypomethylated |
| cg07550016 | 0.695843413 | 0.625382219 | 0.070461194 | 5.04023E-06 | 4 | 0.00105883 | WHSC2 | Body | S_Shore | Hypermethylated |
| cg25726682 | 0.712668402 | 0.652418119 | 0.060250282 | 4.95368E-06 | 1 | 0.001049647 | SLC2A5 | Body | opensea | Hypermethylated |
| cg06281409 | 0.845968146 | 0.899155168 | -0.053187022 | 4.74707E-06 | 5 | 0.001028352 | FBXO38 | Body | opensea | Hypomethylated |
| cg14006863 | 0.312993054 | 0.364220743 | -0.051227688 | 4.58898E-06 | 3 | 0.001012242 | CASR | TSS200 | Island | Hypomethylated |
| cg14339287 | 0.208164406 | 0.12444581 | 0.083718596 | 4.58133E-06 | 19 | 0.001011783 | TECR | TSS200 | Island | Hypermethylated |
| cg18776876 | 0.249064038 | 0.303345235 | -0.054281197 | 4.23124E-06 | 11 | 0.000968431 | TBC1D10C | Body | Island | Hypomethylated |
| cg03625007 | 0.778244334 | 0.724917786 | 0.053326549 | 4.20459E-06 | 2 | 0.000966763 |  | IGR | opensea | Hypermethylated |
| cg25113671 | 0.611430167 | 0.559016587 | 0.05241358 | 3.27328E-06 | 13 | 0.000855783 |  | IGR | opensea | Hypermethylated |
| cg14009504 | 0.59572788 | 0.542262272 | 0.053465607 | 3.18413E-06 | 14 | 0.000843854 | PCNX | Body | opensea | Hypermethylated |
| cg27237398 | 0.660101095 | 0.594409913 | 0.065691182 | 2.78788E-06 | 3 | 0.00078546 | EPHB1 | Body | opensea | Hypermethylated |
| cg02771392 | 0.685117578 | 0.618888401 | 0.066229176 | 2.7226E-06 | 7 | 0.00077822 | ZNF273 | TSS1500 | opensea | Hypermethylated |
| cg19454599 | 0.662772944 | 0.600770635 | 0.062002309 | 2.70494E-06 | 14 | 0.00077614 |  | IGR | opensea | Hypermethylated |
| cg01719123 | 0.212511579 | 0.266831283 | -0.054319704 | 2.38871E-06 | 8 | 0.000731458 | ZNF395 | TSS200 | Island | Hypomethylated |
| cg02826687 | 0.555933927 | 0.500401211 | 0.055532717 | 2.12129E-06 | 8 | 0.00069184 | PMP2 | TSS1500 | opensea | Hypermethylated |
| cg09874802 | 0.671050729 | 0.601365797 | 0.069684932 | 2.0003E-06 | 15 | 0.000670864 |  | IGR | opensea | Hypermethylated |
| cg13508836 | 0.749958325 | 0.690959513 | 0.058998812 | 1.90685E-06 | 8 | 0.000657656 | SCARA5 | TSS1500 | opensea | Hypermethylated |
| cg16855422 | 0.233283175 | 0.288704122 | -0.055420948 | 1.8881E-06 | 11 | 0.000653695 | DNAJC4 | 5'UTR | Island | Hypomethylated |
| cg02267926 | 0.761257713 | 0.69788243 | 0.063375283 | 1.742E-06 | 18 | 0.000622116 | CEP192 | Body | opensea | Hypermethylated |
| cg18895947 | 0.650181408 | 0.593588112 | 0.056593295 | 1.71055E-06 | 2 | 0.000615618 |  | IGR | opensea | Hypermethylated |
| cg13591030 | 0.645835968 | 0.574462157 | 0.071373811 | 1.59769E-06 | 6 | 0.000590917 |  | IGR | opensea | Hypermethylated |
| cg10993701 | 0.446500009 | 0.394020829 | 0.05247918 | 1.45502E-06 | 12 | 0.000562943 | SMAGP | Body | N_Shelf | Hypermethylated |
| cg04515755 | 0.718864467 | 0.660207658 | 0.058656809 | 1.43386E-06 | 1 | 0.00055776 | PRDM16 | Body | opensea | Hypermethylated |
| cg05740992 | 0.672884102 | 0.736896421 | -0.064012319 | 1.21028E-06 | 11 | 0.000507569 |  | IGR | opensea | Hypomethylated |
| cg00437311 | 0.232619898 | 0.283780004 | -0.051160105 | 1.20419E-06 | 19 | 0.000506092 |  | IGR | Island | Hypomethylated |
| cg10787840 | 0.737093197 | 0.66050154 | 0.076591657 | 1.13971E-06 | 14 | 0.000489612 |  | IGR | opensea | Hypermethylated |
| cg17609731 | 0.289926482 | 0.351630423 | -0.061703941 | 1.11622E-06 | 7 | 0.000483888 | RSBN1L | TSS1500 | Island | Hypomethylated |
| cg07129253 | 0.272476859 | 0.324848824 | -0.052371965 | 1.06633E-06 | 8 | 0.000473266 | PURG | TSS1500 | opensea | Hypomethylated |
| cg23550779 | 0.798146368 | 0.738756523 | 0.059389845 | 9.3693E-07 | 12 | 0.000442127 | ERGIC2 | 5'UTR | opensea | Hypermethylated |
| cg14529095 | 0.684999926 | 0.632453326 | 0.0525466 | 9.29818E-07 | 17 | 0.000440828 | PEMT | Body | opensea | Hypermethylated |
| cg18885365 | 0.276998255 | 0.340891819 | -0.063893565 | 8.85322E-07 | 1 | 0.00043095 | MFSD2A | 5'UTR | Island | Hypomethylated |
| cg04570283 | 0.182117875 | 0.237654558 | -0.055536683 | 6.36715E-07 | 14 | 0.000372771 | SEL1L | TSS200 | Island | Hypomethylated |
| cg10059324 | 0.542099728 | 0.473445682 | 0.068654047 | 4.99076E-07 | 1 | 0.000336505 | PER3 | Body | N_Shelf | Hypermethylated |
| cg01986502 | 0.289851795 | 0.349583239 | -0.059731444 | 4.93829E-07 | 4 | 0.000336064 | JADE1 | TSS1500 | Island | Hypomethylated |
| cg16665383 | 0.454902206 | 0.401919635 | 0.052982571 | 4.77664E-07 | 8 | 0.000330856 | C8orf37 | 3'UTR | opensea | Hypermethylated |
| cg25988912 | 0.562637828 | 0.495410231 | 0.067227597 | 4.33181E-07 | 1 | 0.000313725 | LOC101929147 | Body | opensea | Hypermethylated |
| cg16420247 | 0.488491502 | 0.54016295 | -0.051671449 | 4.04886E-07 | 2 | 0.000306446 | HPCAL1 | TSS1500 | Island | Hypomethylated |
| cg26007189 | 0.316328023 | 0.384070486 | -0.067742462 | 4.01848E-07 | 5 | 0.00030591 |  | IGR | N_Shelf | Hypomethylated |
| cg10632328 | 0.295138375 | 0.357176325 | -0.06203795 | 3.47528E-07 | 17 | 0.000286721 | FLJ35220 | Body | Island | Hypomethylated |
| cg25463779 | 0.508033401 | 0.40521325 | 0.102820151 | 2.99447E-07 | 12 | 0.000266935 | FAM101A | TSS1500 | opensea | Hypermethylated |
| cg09601770 | 0.223458709 | 0.281480512 | -0.058021804 | 2.90172E-07 | 2 | 0.000263844 | DPP4 | 1stExon | Island | Hypomethylated |
| cg25024143 | 0.637062934 | 0.581266256 | 0.055796678 | 2.80676E-07 | 17 | 0.000260092 | SP2 | Body | opensea | Hypermethylated |
| cg00488788 | 0.150054853 | 0.098654926 | 0.051399927 | 2.31477E-07 | 15 | 0.000237523 | ONECUT1 | Body | Island | Hypermethylated |
| cg02167717 | 0.883886744 | 0.812267728 | 0.071619016 | 1.99788E-07 | 11 | 0.000224725 | PYGM | Body | S_Shelf | Hypermethylated |
| cg14014565 | 0.749963788 | 0.674480896 | 0.075482893 | 1.89871E-07 | 8 | 0.000217555 | CHD7 | 5'UTR | opensea | Hypermethylated |
| cg00401266 | 0.73318506 | 0.674887089 | 0.058297971 | 1.62055E-07 | 3 | 0.000205663 |  | IGR | opensea | Hypermethylated |
| cg14254882 | 0.74631406 | 0.663555411 | 0.082758649 | 1.55736E-07 | 1 | 0.000203139 |  | IGR | N_Shore | Hypermethylated |
| cg21508212 | 0.729209047 | 0.659081631 | 0.070127417 | 1.47862E-07 | 1 | 0.000198791 | MR1 | TSS1500 | opensea | Hypermethylated |
| cg12723840 | 0.347654229 | 0.399436549 | -0.05178232 | 1.04666E-07 | 1 | 0.000162872 | ATAD3B | TSS200 | Island | Hypomethylated |
| cg13698153 | 0.218654214 | 0.278024472 | -0.059370258 | 8.9028E-08 | 12 | 0.000149753 | VSIG10 | 1stExon | Island | Hypomethylated |
| cg17570835 | 0.573409713 | 0.520194896 | 0.053214817 | 6.36083E-08 | 12 | 0.000131639 | MLXIP | Body | opensea | Hypermethylated |
| cg12543831 | 0.360706563 | 0.411018426 | -0.050311863 | 6.06079E-08 | 8 | 0.000128 | EGR3 | Body | Island | Hypomethylated |
| cg20412217 | 0.700902942 | 0.637474725 | 0.063428217 | 5.53368E-08 | 4 | 0.000122942 | RGS12 | Body | S_Shelf | Hypermethylated |
| cg07205286 | 0.307397906 | 0.24649564 | 0.060902266 | 5.14952E-08 | 5 | 0.000119682 | C5orf66 | TSS1500 | S_Shore | Hypermethylated |
| cg18437077 | 0.314146768 | 0.370936241 | -0.056789474 | 3.28533E-08 | 7 | 9.95747E-05 | PLOD3 | 1stExon | Island | Hypomethylated |
| cg05876035 | 0.710944943 | 0.644113432 | 0.06683151 | 3.17389E-08 | 2 | 9.86655E-05 | LINC01250 | Body | opensea | Hypermethylated |
| cg18645906 | 0.247711127 | 0.299706678 | -0.051995551 | 2.44421E-08 | 4 | 8.87282E-05 | BST1 | 1stExon | N_Shore | Hypomethylated |
| cg01330050 | 0.162743918 | 0.215760886 | -0.053016968 | 9.89499E-09 | 10 | 6.24904E-05 |  | IGR | S_Shore | Hypomethylated |
| cg01535674 | 0.687341434 | 0.623480076 | 0.063861358 | 7.65589E-09 | 7 | 5.60474E-05 |  | IGR | opensea | Hypermethylated |
| cg02353041 | 0.167566195 | 0.229174756 | -0.061608561 | 1.15551E-09 | 9 | 2.35721E-05 | SLC46A2 | TSS200 | Island | Hypomethylated |
| cg24377604 | 0.49118239 | 0.440503371 | 0.050679019 | 8.54148E-10 | 4 | 2.16999E-05 | KIAA1211 | Body | Island | Hypermethylated |
| cg24675448 | 0.287981701 | 0.220441037 | 0.067540664 | 3.30870E-11 | 6 | 2.43769E-06 | EYA4 | 5'UTR | Island | Hypermethylated |
